# Maternal stress in pregnancy and child autism spectrum disorder: evaluating putative causal associations using a genetically informed design

**DOI:** 10.1192/bjo.2021.114

**Published:** 2021-06-18

**Authors:** Mohamed Essam Gamil Abdelrazek, Frances Rice

**Affiliations:** 1 Cardiff Univeristy; 2 Wolfson Centre for Young People's Mental Health, Section of Child and Adolescent Psychiatry, Division of Psychological Medicine and Clinical Neurosciences, Cardiff University

## Abstract

**Aims:**

Prenatal adversity is hypothesized to increase risk of Autism Spectrum Disorder (ASD) via epigenetic changes. Maternal stress in late pregnancy may alter offspring neurodevelopmental outcomes by disrupting a unique period of rapid neurogenesis. Observational studies reporting an environmentally mediated programming pathway face challenges in drawing causal inferences including passive gene-environment correlation. This project aims to use a quasi-experimental genetically informed design to assess if reported correlations between maternal prenatal stress and offspring ASD traits were due to maternally inherited factors or consistent with a potentially causal prenatal exposure effect. No previous cross-fostering studies have assessed the effects of prenatal stress on childhood ASD.

**Method:**

This study used an in-vitro fertilization cross-fostering sample with pregnant mothers related (n = 365) or unrelated (n = 111) to their offspring (mean age = 9.84 years). Prenatal stress was assessed using a subjective Likert scale during pregnancy. Questionnaires examined maternally rated offspring ASD traits using the Social and Communication Disorders Checklist. Birth weight and gestational age from medical records were used as comparison outcomes to validate the measure of stress as evidence suggests they are influenced by environmental factors. Correlations from multiple regression models were examined in relation to magnitude of effect size as well as significance. This is partly due to small sample size and that cross-fostering designs rely on comparing magnitudes of associations between related and unrelated groups. An interaction term was used to test the difference in the strength of association between related and unrelated mother-child groups.

**Result:**

Subjective assessment of prenatal maternal stress showed construct validity as it was associated with low birth weight (β = –0.297, p = 0.005) and reduced gestational age (β= –0.320, p = 0.001). Subjective late pregnancy stress was associated with increased offspring ASD traits in the whole sample (β = 0.089, p = 0.073) and in the related (β=0.045, p = 0.424) and unrelated mother-child (β=0.233, p = 0.029) subgroups. Non-significant interaction terms demonstrated that the mechanisms underlying the association between maternal stress and ASD and birth outcomes are likely to be similar and environmentally driven in the different conception groups.

**Conclusion:**

Findings demonstrate the utility of genetically informed designs in disentangling inherited factors from environmental influences in the study of prenatal risk factors. Correlations between maternal prenatal stress and offspring ASD being present in both related and unrelated mother-child groups indicate an environmental link that is consistent with a potential causal effect. Associations detected are of imperative use for clinicians and policymakers, as they can guide the implementation of early psychosocial care for families at high liability.

